# Draft Genome Sequencing of the Highly Halotolerant and Allopolyploid Yeast *Zygosaccharomyces rouxii* NBRC 1876

**DOI:** 10.1128/genomeA.01610-16

**Published:** 2017-02-16

**Authors:** Atsushi Sato, Kenichiro Matsushima, Kenshiro Oshima, Masahira Hattori, Yasuji Koyama

**Affiliations:** a Research & Development Division, Kikkoman Corporation, Noda, Chiba, Japan; b Noda Institute for Scientific Research, Noda, Chiba, Japan; c Graduate School of Frontier Sciences, University of Tokyo, Kashiwa, Chiba, Japan; d Graduate School of Advanced Science and Engineering, Waseda University, Shinjuku-ku, Tokyo, Japan

## Abstract

The highly halotolerant and allopolyploid yeast *Zygosaccharomyces rouxii* is industrially used for the food production in high concentrations of salt, such as brewing soy sauce and miso paste. Here, we report the draft genome sequence of *Z. rouxii* NBRC 1876 isolated from miso paste.

## GENOME ANNOUNCEMENT

*Zygosaccharomyces rouxii* is an osmotolerant and halotolerant yeast that grows in high concentrations of salt and/or sugar (1). This yeast is industrially used for brewing soy sauce and miso paste in high concentrations of salt (2).

It is supposed that there are at least two different genomic types in *Z. rouxii*. One is haploid type with one copy of each gene, which includes CBS 732^T^ isolated from concentrated black grape must (3). Another is allopolyploid hybrids, which includes ATCC 42981 isolated from miso paste, with two copies of functional genes involved in the production of glycerol as a compatible solute to protect the cell against lysis and efflux of Na^+^ from cells in high concentrations of salt (4–7). These redundant genes in an allopolyploid strain can contribute to survival under high-osmotic conditions, such as the process of brewing soy sauce. In soy sauce brewing, *Z. rouxii* contributes to the production of its distinctive aroma (8).

The genomic DNA of the allopolyploid type has been partially sequenced (7) but not enough to analyze the genome in detail, while that of the haploid type strain was completely sequenced (9). Therefore, we conducted genome sequencing of *Z. rouxii* NBRC 1876, isolated from miso paste, as a model strain of osmotolerant/halotolerant yeast with an allopolyploid genome.

Cultivation of NBRC 1876 and genomic DNA extraction that followed were performed according to the methods described in our previous report (10). The genomic DNA was sequenced using Roche 454 GS FLX+ single-end and titanium paired-end sequencing. To remove artificial replicates from emulsion PCR, all single-end reads were identified using CD-HIT-454 (11). All paired-end reads were confirmed longer than 45-mer sequences. After removing all artifacts, the obtained reads were assembled using the GS *de novo* assembler (12). The assembly generated 62 nonredundant scaffolds composed of 482 contigs, and the total genome size was estimated to be 19.4 Mb, which was twice that of CBS 732 (9.8 Mb) (9). This result suggests that NBRC 1876 is allopolyploid. This genome information contributes to further studies on food science and of its physiology and taxonomy.

### Accession number(s).

This whole genome shotgun project has been deposited in DDBJ/ENA/GenBank. Accession numbers for the 62 scaffold sequences are DF983528 to DF983589. Accession numbers for the 482 contig sequences are BBQV01000001 to BBQV01000482. The version described in this paper is the first version, BBQV01000000.
